# Angiopoietin-1 deficiency increases tumor metastasis in mice

**DOI:** 10.1186/s12885-017-3531-y

**Published:** 2017-08-11

**Authors:** Iacovos P. Michael, Martina Orebrand, Marta Lima, Beatriz Pereira, Olga Volpert, Susan E. Quaggin, Marie Jeansson

**Affiliations:** 1 Swiss Institute for Experimental Cancer Research, School of Life Sciences, Swiss Federal Institute of Technology Lausanne (EPFL), Lausanne, Switzerland; 2 Department of Immunology, Genetics and Pathology, Uppsala University, Dag Hammarskjoldsvagen 20, 751 85 Uppsala, Sweden; 3 Matrix Dynamics Group, Faculty of Dentistry, University of Toronto, Toronto, Canada; 4 Department of Urology, RH Lurie Comprehensive Cancer Center, Northwestern University, Chicago, IL USA; 5 Feinberg Cardiovascular Research Institute and Division of Nephrology and Hypertension, Northwestern University, Chicago, IL USA

**Keywords:** Angiopoietin-1, Metastasis, MMTV-PyMT, B16F10 melanoma

## Abstract

**Background:**

Angipoietin-1 activation of the tyrosine kinase receptor Tek expressed mainly on endothelial cells leads to survival and stabilization of endothelial cells. Studies have shown that Angiopoietin-1 counteracts permeability induced by a number of stimuli. Here, we test the hypothesis that loss of Angiopoietin-1/Tek signaling in the vasculature would increase metastasis.

**Methods:**

Angiopoietin-1 was deleted in mice just before birth using floxed Angiopoietin-1 and Tek mice crossed to doxycycline-inducible bitransgenic ROSA-rtTA/tetO-Cre mice. By crossing Angiopoietin-1 knockout mice to the MMTV-PyMT autochthonous mouse breast cancer model, we investigated primary tumor growth and metastasis to the lung. Furthermore, we utilized B16F10 melanoma cells subcutaneous and experimental lung metastasis models in Angiopoietin-1 and Tek knockout mice.

**Results:**

We found that primary tumor growth in MMTV-PyMT mice was unaffected, while metastasis to the lung was significantly increased in Angiopoietin-1 knockout MMTV-PyMT mice. In addition, angiopoietin-1 deficient mice exhibited a significant increase in lung metastasis of B16F10 melanoma cells, compared to wild type mice 3 weeks after injection. Additional experiments showed that this was likely an early event due to increased attachment or extravasation of tumor cells, since seeding of tumor cells was significantly increased 4 and 24 h post tail vein injection. Finally, using inducible Tek knockout mice, we showed a significant increase in tumor cell seeding to the lung, suggesting that Angiopoietin-1/Tek signaling is important for vascular integrity to limit metastasis.

**Conclusions:**

This study show that loss of the Angiopoietin-1/Tek vascular growth factor system leads to increased metastasis without affecting primary tumor growth.

## Background

The angiopoietin/Tek system has become a target of growing interest in the development of cancer therapeutics [1]. Angiopoietin-1 (Angpt1) is an activator of tyrosine kinase receptor Tek (also called Tie2) expressed mainly on endothelial cells. Tek activation and phosphorylation results in downstream signaling promoting vascular maturity and endothelial cell survival [2]. Angiopoietin-2 (Angpt2) antagonizes Angpt1 binding and thus Tek signaling in endothelial cells [3], but can also act as a weak agonist in the absence of Angpt1 [4]. Angpt2 may also have an agonistic role in lymphatic vessels [5, 6]. Angpt2 can activate integrins, leading to endothelial destabilization [7], an effect that may be increased in situations of low Tek expression [8]. In regards to vascular leakage, Angpt1 counteracts hyperpermeability induced by several leakage promoting stimuli [9, 10] while Angpt2 weakens the vascular barrier [11, 12].

It is known that Angpt2 is elevated in many human cancers [2] and preclinical studies of anti-Angpt2 agents often demonstrate additive anti-angiogenic effects on primary tumor growth when combined with inhibitors of the VEGF pathway (reviewed in [1]). Furthermore, Angpt2 blocking antibody has been shown to inhibit metastatic dissemination to the lung in part by enhancing endothelial cell-cell junction integrity [13]. Increased Tek activation has been shown to decrease metastasis in mice in different experimental models of cancer [14–16]. Wu et al. tested the anti-metastatic potential of vasculotide, a purported Angpt1 mimetic, in experimental metastasis. Tumor cells were injected directly into the venous circulation thus modelling the late stages of metastasis, i.e. extravasation and seeding into distant organ. Vasculotide reduced human breast cancer cell extravasation to the lung, but failed to inhibit human colon cancer extravasation to liver and lymphatics as well as human renal cancer cell extravasation to lung [14]. Interestingly, vasculotide also delayed dissemination of spontaneous lung metastases from orthotopic breast cancer xenographs without affecting primary tumors [14]. Goel et al. showed anti-metastatic effects in vivo utilizing an inhibitor of vascular endothelial protein tyrosine phosphatase (VE-PTP), AKB-9778 [15]. Normally, VE-PTP deactivates Tek, thus an inhibitor of VE-PTP would sustain Tek activation. VE-PTP inhibition delayed the early phase of mammary tumor growth and slowed growth of lung metastases. Park et al., recently showed that administration of an antibody, ABTAA (Angpt2-binding and Tek activating antibody), resulted in normalization of tumor vessels, reduced tumor growth, reduced metastasis, and enhanced drug delivery [16]. In contrast, adenoviral overexpression of Angpt1 in mice facilitated tumor cell dissemination and metastasis establishment [17].

In the clinic, the dual Angpt2 and Angpt1-neutralizing peptibody, trebananib (AMG386), recently failed to improve overall survival when combined with paclitaxel in patients with recurrent platinum-sensitive ovarian cancer in the phase III TRINOVA-1 trial, despite earlier improved progression-free survival [18]. Trebananib also failed in phase II trials involving metastatic gastro-esophageal [19], colorectal [20], and metastatic clear cell renal carcinomas [21]. Although Trebananib has a higher affinity for Angpt2 compared to Angpt1, the inhibition of Angpt1 is thought to contribute to its lack of effect. It is evident that more studies are needed to define how Angpt1, Angpt2 and Tek act in tumor growth and what their roles are in metastasis in order to develop better therapies.

To clarify the role of Angpt1 and Tek in tumor metastasis, we utilized doxycycline-inducible conditional Angpt1 and Tek knockout mice. We investigated how Angpt1 deficiency affected tumor growth and lung metastasis by crossing these mice to the MMTV-PyMT transgenic mice that develop mammary tumors and lung metastasis. To investigate the initial phase of metastasis, extravasation, we also performed intravenous injection of tumor cells in Angpt1 and Tek deficient mice to evaluate dissemination to the lung. Overall, we found that loss of Angpt1/Tek leads to increase distant metastasis without affecting primary tumor growth.

## Methods

### Mice & breeding

Floxed Angpt1 and floxed Tek mice, were crossed with a ROSA-rtTA/tetO-Cre bitransgenic mice to generate inducible whole body knockout of *Angpt1* (*Angpt1* ^*Δ/Δ*^) or *Tek* (*Tek* ^*Δ/Δ*^) upon administration of doxycycline in the drinking water as previously described [6, 22]. In short, knockout was induced at embryonic day 16.5 by administration of doxycycline as above in the pregnant dam’s drinking water until weaning. Controls (WT) were Angpt1 *w*/w or Tek *w*/w littermates with ROSA-rtTA, tetO-Cre. All mice received doxycycline.

Transgenic mice expressing polyomavirus middle T (PyMT) oncogene under control of the mouse mammary tumor virus long terminal repeat (MMTV) [23] were crossed with *Angpt1* ^*Δ/Δ*^, all mice were backcrossed >6 generations to FVB. Both male and female (50/50) mice on a mixed background and 8–12 weeks old were used for all other experiments. Littermate mice negative for floxed alleles were used as controls (WT). All animal experiments were approved by the local ethics review committees of Mount Sinai Hospital (Toronto, ON, Canada), Northwestern University (Chicago, IL), and Uppsala University (Sweden, ethical # C122412/13 and C99/15).

Mice were genotyped by PCR using the following primer pairs; Angpt1 flox (for 5′-CAATGCCAGAGGTTCTTGTGAA and rev 5′-TCAAAGCAACATATCATGTGCA, Angpt1 wt 233 bp, flox 328 bp), Angpt1 del (for 5′-CAATGCCAGAGGTTCTTGTGAA and rev 5′-TGTGAGCAAAACCCCTTTC, 481 bp), ROSA-rtTA (for 5′-GAGTTCTCTGCTGCCTCCTG and rev 5′-AGCTCTAATGCGCTGTTAAT), general Cre allele (for 5′-ATGTCCAATTTACTGACCG and rev 5′-CGCCGCATAACCAAGTGAA, 673 bp), Tek wt (for 5′- TCCTTGCCGCCAACTTGTAAAC and rev 5′- AGCAAGCTGACTCCACAGAGAAC, 175 bp), Tek flox (for 5′- TCCTTGCCGCCAACTTGTAAAC and rev 5′- AGCAAGCTGACTCCACAGAGAAC, 604 bp) and PyMT (for 5′- GGAAGCAAGTACTTCACAAGGG and rev 5′- GGAAAGTCACTAGGAGCAGGG, 530 bp).

### Tumor growth and metastasis in MMTV-PyMT-transgenic mice

Female 6-week old MMTV-PyMT-*Angpt1* ^*Δ/Δ*^ (*n* = 4), MMTV-PyMT-WT (*n* = 5), *Angpt1* ^*Δ/Δ*^ (*n* = 7) and WT (*n* = 10) mice were used in the study. All MMTV- PyMT mice were heterozygous for MMTV-PyMT. Bodyweight was recorded weekly and mice were euthanized at 16 weeks of age. Weight and volume of individual mammary tumors were measured. Tumor volume was calculated using the formula V = (L x W x W)/2. Tumors and lungs were fixed in 10% formalin for 4 h and then embedded in paraffin. Paraffin sections from 4 levels of the lungs were stained with rat-anti-PyMT (Santa Cruz) and scanned using NanoZoomer (Hamamatsu). Lung tumors were counted and the tumor area was calculated from the measured diameter of individual tumors and compared to total lung area using NanoZoomer software (Hamamatsu).

### B16F10 melanoma transfection and characterization

B16F10 melanoma cells (ATCC) were maintained in DMEM (10% FCS, 2 mM L-glutamine, 100 U/ml penicillin/streptomycin) at 37°C in a humidified 5% CO_2_ incubator. For the generation of stable EGFP expressing B16F10 melanoma cell, 1 × 10^6^ cells were plated per well (9.6 cm^2^/well) in a 6-well plate and transfected 16 h after with 45 μg of PB-EGFP along with 1 μg PBase using ExGen500 (R0511, Fermentas) according to the manufacturer’s protocol. Stable clones were derived after 2 weeks of selection using 1 μg/ml puromycin. Cells were then trypsinized and sorted by FACS to collect the 10% of cells with highest GFP signal; these cells were further expanded for experiments. Cells were positively identified as melanoma cells by their deposits of melanin in vitro and in vivo. Cells were tested negative for *Mycoplasma*, and cells were typically used in experiments within 4 passages after thawing.

Gene expression analysis was done on B16F10 cells to investigate if they express components of the Angpt/Tek system and compared to expression in lung of adult wildtype mice. Trizol (Invitrogen) was used to extract mRNA according to the manufacturer’s protocol, followed by cDNA synthesis using iScript reverse transcription supermix (BioRad). Real time PCR was performed using 100 ng of cDNA with iTaq universal SYBR Green supermix (BioRad) and appropriate primers on a CFX-96 Real Time system (BioRad). Expression results were normalized to endogenous control *Hprt* and relative quantification was done using the Livak method (2^-ΔΔCT^) [24]. The following primer pairs were used for analysis; *Hprt* (for 5′- GGCTATAAGTTCTTTGCTGACCTG and rev 5′- AACTTTTATGTCCCCCGTTGA), *Angpt1* (for 5′- GGGGGAGGTTGGACAGTAA and rev 5′- CATCAGCTCAATCCTCAGC), *Angpt2* (for 5′- GATCTTCCTCCAGCCCCTAC and rev 5′- TTTGTGCTGCTGTCTGGTTC), *Tek* (for 5′- TGGAGTCAGCTTGCTCCTTT and rev 5′- ACCTCCAGTGGATCTTGGTG), *Vegfa* (for 5′- CAGGCTGCTGTAACGATGAA and rev 5′- CTATGTGCTGGCTTTGGTGA) and *Tgfb1* (for 5′- TGAGTGGCTGTCTTTTGACG and rev 5′- CGCACACAGCAGTTCTTCTC).

To investigate if Angpt1 could affect migration and behavior of B16F10 melanoma cells we performed in vitro studies. B16F10 cells (40,000 and 20,000 cells) were seeded in a 96-well plate and allowed to grow O/N at the same conditions as above. A scratch was made in each well using Woundmaker (Essen Bioscience), and 2000 ng/ml of Angpt1 (130–06, Peprotech) was used to stimulate half of the wells. Wells were imaged every 20 min for 24 h using Incucyte Zoom (Essen Bioscience) and migration was calculated using the manufacturer’s software.

### B16F10 Melanoma tumor growth and metastasis

To study primary tumor growth, 1 × 10^6^ B16F10 cells was injected subcutaneously (s.c.) at 2 sites on the flank of 7 *Angpt1* ^Δ/Δ^ mice and 5 WT mice. Tumors were measured with calipers to calculate tumor volume and mice were euthanized 15 days after injection.

To study tumor metastasis, 1 × 10^5^ B16F10 cells were injected in the dorsal tail vein of 20 *Angpt1* ^Δ/Δ^ mice and 19 WT mice. Mice were euthanized 21 days after injection, and lungs were fixed and cut in 1 mm sections were tumors were counted using a dissection microscope. A similar experiment was done in 14 *Tek* ^Δ/Δ^ mice with 16 WT C57 mice as controls. Earlier time points were also used where 2 × 10^6^ B16F10 cells were injected and tissue harvested after 24 h in *Angpt1* ^Δ/Δ^ and WT mice (*n* = 6 for both groups). At the 24 h time point lungs were viewed at 40× using a fluorescence stereo zoom microscope (AZ100, Olympus). GFP-positive cells were quantified from images using Elements software.

Vascular leakage was evaluated using cadaverine (~1 kD) conjugated to Alexa Fluor 555 (Thermo Fisher Scientific). Cadaverine (0.1 mg) was injected via the tail vein in two groups of *Angpt1* ^Δ/Δ^ mice and two groups of WT mice (*n* = 4 for each group). Cadaverine was allowed to circulate for 10 min before injection of 1 × 10^6^ B16F10 cells in one *Angpt1* ^Δ/Δ^ group and one WT group. After 4 h, all 4 groups of mice were perfused with HBSS to clear out cadaverine in blood vessels. One lobe was weighted and homogenized in PBS, followed by measurement of fluorescent signal in a plate reader. Other parts of the lungs were fixed and imaged in a dissection microscope at 60× followed by quantification of GFP-positive pixels using Adobe Photoshop.

Integrin signaling was investigated in another set of experiments. Lungs from WT and *Angpt1* ^Δ/Δ^ at baseline and 4 h after tail vein injection of 1 × 10^6^ B16F10 cells were dissected and studied. Protein from lungs was extracted by homogenizing tissue in RIPA buffer (Thermo Fisher Scientific) containing protease and phosphatase inhibitors (PhosSTOP and Complete, Roche). Following incubation at 4°C and centrifugation, supernatant was collected and measured for protein concentration using a BCA assay (Pierce), aliquoted and stored at −80°C. For Western blot analysis, 20 μg denatured protein samples were separated on 4–20% MiniProtean gels (BioRad) and then transferred to PVDF membranes. Blots were blocked with 5% BSA and incubated with primary antibodies, rabbit anti-pFAK(397) (44-625G, Thermo Fisher Scientific) and rabbit-anti-β-actin (4967, Cell Signaling). After washing and incubation with anti-rabbit HRP-conjugated secondary antibody, proteins were visualized using ECLplus detection reagents (GE, Uppsala, Sweden).

### Macrophages

Macrophages in the lung were evaluated by FACS 3 weeks after tail vein injection of 1 × 10^5^ B16F10 cell in *Angpt1* ^Δ/Δ^ and WT mice. Lungs were dissected and digested into single cell suspension by incubation in Hank’s Balanced Salt Solution (HBSS) containing 1 mg/ml Collagenase I, 1 mg/ml Collagenase IV, 1 mg/ml Collagenase V, and 1 U/ml DNAse I for 45 min at 37°C. The cells were then washed 3 times by centrifugation at 500 x g for 5 min and exchange of buffer, HBSS with 3% BSA and 1 mM EDTA. Lung suspensions were stained with anti-mouse CD36 (BD Bioscience) for macrophages and anti-mouse CD206 (BD Bioscience) to identify changes in macrophage polarization between *Angpt1* ^Δ/Δ^ and WT mice in a BD FACS Calibur. In addition, staining with anti-Tek (124,007, Biolegend) was done. Mean fluorescence intensity (MFI) was calculated using BD FlowJo software (BD Bioscience). Quantification of macrophages labelled with Isolectin B4 was done on vibratome sections of lung using a Leica SP8 confocal microscope.

### Electron microscopy and Microsphere experiments

To investigate if the diameter of lung capillaries were different in *Angpt1* ^Δ/Δ^ mice at baseline we utilized performed measurements on micrographs and studied the distribution of microspheres in WT and *Angpt1* ^Δ/Δ^ mice.

For electron microscopy, lungs from 4 *Angpt1* ^Δ/Δ^ and 4 WT mice were harvested, cut in 1 mm cubes, and immersion fixed in Karnovsky’s fixative (2.5% paraformaldehyde and 2% glutaraldehyde in 0.05 M Na-cacodylate buffer pH 7.2). Tissue were post fixed in 1% OsO4 for 1 h, dehydrated in alcohol and embedded in epoxy resin and heat-cured. Ultrathin sections (~50 nm) were contrasted with lead citrate and uranyl acetate and examined in a Tecnai G2 electron microscope. Micrographs were taken at 4200× and capillaries defined as a vessel containing 1 red blood cell. At least 50 micrographs were taken from each animal and the cross sectional area of capillaries was measured using ImageJ. An average was calculated from each animal which was then used to calculate the group average.

To investigate microsphere distribution, 1 × 10^5^ fluorescent microspheres (FluoSpheres, Invitrogen) were injected in the dorsal vein of 5 WT and 5 *Angpt1* ^Δ/Δ^ mice. The microspheres had a diameter of ~15 μm which is similar to B16F10 melanoma cells. Mice were euthanized 4 h after injection, and lungs were fixed O/N at 4 °C in 10% formalin. Vibratome sections of 100 μm thicknesses were counterstained, mounted and imaged in a confocal microscope (SP8, Leica). The number of microspheres was counted on tile scan images of whole lung sections using ImageJ and then expresses as microspheres/area lung tissue.

### Attachment assay of B16F10 melanoma cells

To investigate if the attachment of B16F10 cells to endothelial cells could be affected by Angpt1 we performed attachment assays. Human umbilical vein endothelial cells (HUVECs; passage 5–7) were routinely cultured in gelatin-coated tissue culture flasks in EGM-MV medium. For experiments, HUVECs were seeded in 24-well plates (50,000 cells/well) and allowed to reach >90 confluency. HUVECs were pre-incubated with 1000 ng/ml Angpt1 (ALX-201-314-C050, Enzo Life Sciences) for 4 h before adding B16F10 melanoma cells to wells (10,000 cells/well). The control experiments (Angpt1-) were performed without Angpt1. Cells were then incubated at 37°C with occasional movement of the plate for 10 min. Cells were then washed carefully twice with PBS to remove unattached B16F10 cells, fixed in 10% formalin and stained with Hoechst 33,258. Each well was imaged and GFP positive cells (B16F10 cells) were quantified. Experiments were performed 8 times with four replicates for both conditions.

### RNA sequencing data

Extraction of RNA-seq data was done for two different datasets to investigate gene expression. A published study was utilized to look at expression levels in late stage carcinoma tumors of mammary glands from MMTV-PyMT mice compared to FVB mammary gland [25]. The data was downloaded from NCBI GEO database (accession number: GSE76772).

We also extracted data from RNA-seq experiments from the lungs of adult WT (*n* = 3) and *Angpt1* ^Δ/Δ^ (*n* = 4) mice (unpublished data, Jeansson lab). In these experiments, a cDNA library was made using SMARTer Stranded Total RNA Sample Prep Kit (Clontech). Sequencing was performed on an Illumina HiSeq 2500.

### Statistical Analysis

Data are expressed as mean ± SEM unless otherwise stated. Statistical analysis was performed using 2-tailed Student’s t-test to analyze statistically significant differences between groups. Logarithmic values were used in the case of a skewed distribution. A *p* < 0.05 is considered to be statistically significant.

## Results

### Angiopoietin-1 deficiency enhances lung metastasis without affecting primary tumor growth

**Fig. 1:**
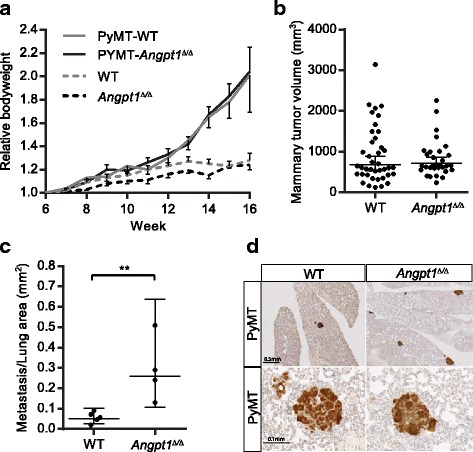
*Angpt1* deficiency increases lung metastasis in MMTV-PyMT mammary tumor model. **a** Bodyweight ± SEM from 6 weeks until 16 weeks of age for female MMTV-PyMT-*Angpt1* ^*Δ/Δ*^ (*n* = 4), MMTV-PyMT-WT (*n* = 5), *Angpt1* ^*Δ/Δ*^ (*n* = 7) and WT (*n* = 10) mice. **b** Mean ± 95% CI for mammary tumor volumes was similar in MMTV-PyMT- *Angpt1* ^*Δ/Δ*^ and MMTV-PyMT-WT mice at 16 weeks. **c** Mean ± 95% CI for metastasis/lung area show a significant increase in metastasis in MMTV-PyMT-*Angpt1* ^*Δ/Δ*^ compared to MMTV-PyMT-WT mice. **p < 0.01. **d** Lungs with tumors stained for MMTV-PyMT

To determine if Angpt1 plays a role in metastasis we used inducible whole body Angpt1 (*Angpt1* ^*Δ/Δ*^) knockout mice crossed with the MMTV-PyMT transgenic mouse model of mammary tumors and lung metastasis. All MMTV-PyMT positive mice developed mammary tumors, and the body weight was significantly increased in both MMTV-PyMT- *Angpt1* ^*Δ/Δ*^ and MMTV-PyMT-WT mice compared to MMTV-PyMT negative mice (Fig. 1a). Endpoint measurements of mammary tumor volume and tumor weight were similar in MMTV-PyMT- *Angpt1* ^*Δ/Δ*^ mice and MMTV-PyMT-WT mice (Fig. 1b, data not shown). All MMTV-PyMT mice had metastasis to the lung, but there was a significant (*p* < 0.01) increase in MMTV-PyMT-*Angpt1*KO mice compared to MMTV-PyMT-WT mice (Fig. 1c, d).

**Fig. 2:**
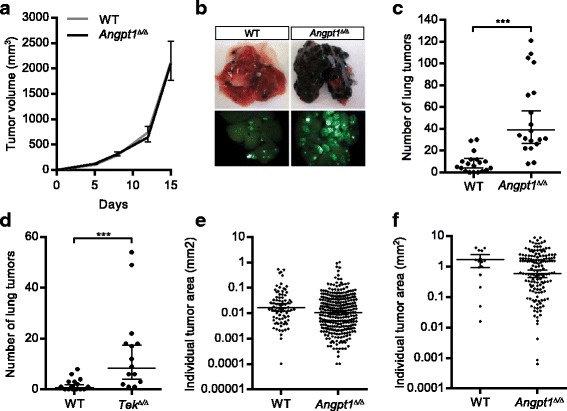
Angpt1 deficiency increases lung metastasis of B16F10 melanoma experimental metastasis model. **a** Mean ± SEM for B16F10 subcutaneous tumor volume over 15 days comparing WT (*n* = 4) and *Angpt1* ^*Δ/Δ*^ (*n* = 7) mice. **b**, **c** Quantification of lung metastasis of B16F10 melanoma cells 3 weeks after tail vein injection in *Angpt1* ^*Δ/Δ*^ mice (*n* = 20) compared to WT mice (*n* = 19). **d** Quantification of lung metastasis of B16F10 melanoma cells 3 weeks after tail vein injection in *Tek* ^*Δ/Δ*^ mice (*n* = 14) compared to WT mice (*n* = 15). Data is shown as mean ± 95% CI. ***p < 0.001. **e**, **f** Size of lung metastatic foci in WT and *Angpt1* ^*Δ/Δ*^ MMTV-PyMT mice (**e**) and B16F10 experimental metastasis model (**f**). Data is shown as mean ± 95% CI

To further study tumor growth and metastasis in *Angpt1* ^*Δ/Δ*^ mice we utilized B16F10 melanoma cells. In a first set of experiments, B16F10 cells were injected subcutaneously to study primary tumor growth. Just as in the MMTV-PyMT experiments there was no difference in primary tumor growth when comparing *Angpt1* ^*Δ/Δ*^ mice and WT mice (Fig. 2a). We then performed experimental metastasis assays; B16F10 cells were injected in the tail vein which resulted in significantly (*p* < 0.001) more lung metastatic foci 3 weeks after injection in *Angpt1* ^*Δ/Δ*^ mice compared to WT (Fig. 2b, c). To investigate if these results were dependent on Tek signaling we also performed B16F10 tail vein injections in *Tek* ^*Δ/Δ*^ mice. Similar to *Angpt1* ^*Δ/Δ*^ mice, *Tek* ^*Δ/Δ*^ mice also had a significant (*p* < 0.001) increase in lung tumors 3 weeks after tail vein injection of B16F10 melanoma cells (Fig. 2d). Individual tumor area of lung metastases from MMTV-PyMT experiments and B16F10 experiments were not different comparing WT and *Angpt1* ^*Δ/Δ*^ mice (Fig. 2e, f).

### Macrophage polarization is not affected in *Angpt1*^*Δ/Δ*^ mice

**Fig. 3:**
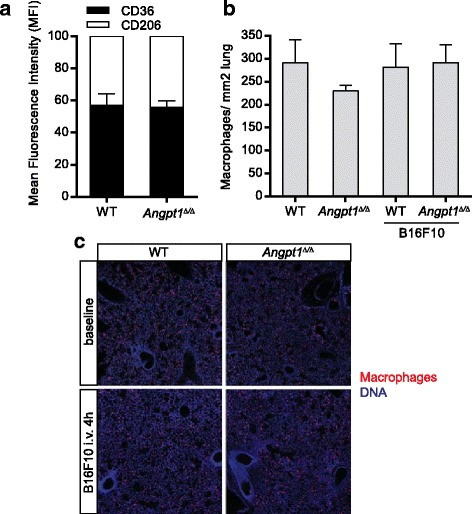
Macrophage polarization is not affected in Angpt1 deficient. Individual lung metastases sizes were not different between WT and *Angpt1* ^*Δ/Δ*^ in MMTV-PyMT mice (**a**) and B16F10 i.v melanoma injections (**b**), data is shown as mean ± 95% CI. (**c**) (**a**) FACS analysis of CD36 and CD206 expression in lung macrophages 3 weeks after B16F10 in WT and *Angpt1* ^*Δ/Δ*^ mice. **b**, **c** Quantification of total number of macrophages did not show any differences at baseline or 4 h after B16F10 melanoma cell injection

Angpt1 has several well-known anti-inflammatory properties and Angpt2 has been shown to activate Tek-positive tumor associated macrophages [26]. We therefore investigated if changes in macrophages contributed to increased metastasis in *Angpt1* ^*Δ/Δ*^ mice. Changes in macrophage polarization were assessed using FACS of lung tissue 3 weeks after tumor cell injection in *Angpt1* ^*Δ/Δ*^ and WT mice. We found no difference in macrophage polarization comparing *Angpt1* ^*Δ/Δ*^ and WT mice (Fig. 3a).The proportion of Tek-positive macrophages was 12.4 ± 2.0% in WT mice and 15.6 ± 1.0% in *Angpt1* ^*Δ/Δ*^ mice (ns). The total number of macrophages was also similar (Fig. 3b, c).

### Angiopoietin-1 deficiency enhances the initial seeding of tumor cells in the lungs

**Fig. 4:**
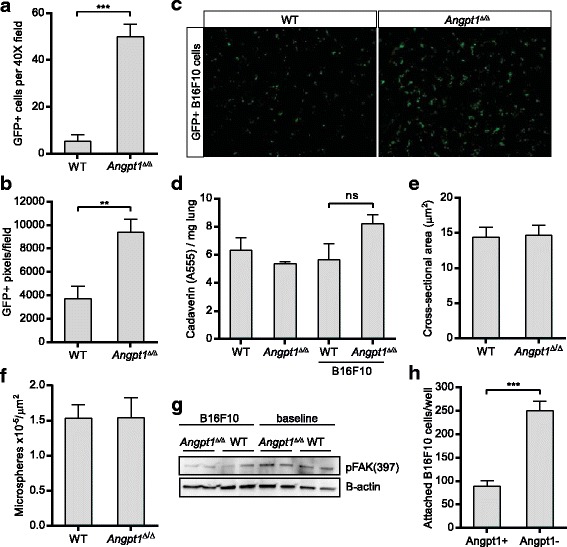
Angpt1 deficiency enhances initial stages of distant metastasis. **a** Quantification of B16F10 cells in the lungs 24 h after tail vein injection in WT (*n* = 6) and *Angpt1* ^*Δ/Δ*^ mice (*n* = 6). **b** Quantification of B16F10 cells in the lungs 4 h after tail vein injection in WT and *Angpt1* ^*Δ/Δ*^ mice (*n* = 4–5). **c** Representative fluorescent image of lung from WT and *Angpt1* ^*Δ/Δ*^ mice 4 h after tail vein injection of GFP positive B16F10 cells. **d** Leakage experiments with cadaverin in WT and *Angpt1* ^*Δ/Δ*^ mice, with and without tail vein injection of B16F10 cells. **e** Measurement of cross-sectional area of capillaries from micrographs of lungs from WT (*n* = 4) and *Angpt1* ^*Δ/Δ*^ (*n* = 4) mice. **f** Quantification of microspheres in lungs 4 h after tail vein injection in WT (*n* = 5) and *Angpt1* ^*Δ/Δ*^ (*n* = 5) mice**p < 0.01, ***p < 0.001. **g** Western blotting for FAK phosphorylation at Tyr397 in WT and *Angpt1* ^*Δ/Δ*^ mice, with and without tail vein injection of B16F10 cells. **h** Quantification of B16F10 cell attachment to HUVECs with or without pre-treatment with Angpt1

Tail vein injections of B16F10 cells were used to further examine the tumor seeding difference in the lung. Quantifications of tumor cells in lung 4 h and 24 h after injection revealed a significant (*p* < 0.01 and *p* < 0.001, respectively) increase in tumor cells in *Angpt1* ^*Δ/Δ*^ mice compared to WT mice (Fig. 4a-c). At the 4 h time point tumor cells are most likely still in the vessels lumen. At later time points it was not possible to determine if the tumor cells had extravasated or not.

To understand the mechanism of enhanced seeding to the lungs of *Angpt1* ^*Δ/Δ*^ mice, we first assessed whether increased vascular leakage could be the reason. Previous studies have not shown any vascular leakage in *Angpt1* ^*Δ/Δ*^ mice at baseline [6, 22]; however, this could be a possibility in the presence of tumor cells. Cadaverine was injected intravenously followed by injection of B16F10 cells to measure vascular leakage in WT and *Angpt1* ^*Δ/Δ*^ mice. As expected there was no difference in leakage at baseline comparing WT and *Angpt1* ^*Δ/Δ*^ mice, and no significant changes could be seen after B16F10 injection either (Fig. 4d).

Goel et al. recently showed that inhibition of VE-PTP increased Tek activity and inhibited several stages of tumor progression and metastasis [15]. Apart from structural and functional normalization of tumor vessels VE-PTP inhibition resulted in an increase in vessel diameter leading to improved tumor perfusion and reduced hypoxia [27]. A gain-of-function mutation of Tek has been identified in patients with venous malformations [28] and several experimental treatments to increase Tek signaling show increased vessel diameter and blood flow [29, 30]. It is possible a change in capillary diameter could change the interaction between vascular endothelial cells and B16F10 cells, thus increasing attachment and extravasation. We therefore investigated cross-sectional area of lung capillaries and found it to be similar in *Angpt1* ^*Δ/Δ*^ mice and WT mice (Fig. 4e). To further study if a smaller capillary diameter could explain the increased seeding of tumor cells to the lung we injected microspheres of similar size (∅15 μm) as the tumor cells intravenously. Microspheres showed a similar distribution in WT and *Angpt1* ^*Δ/Δ*^ mice (Fig. 4f)

It has been described that Angpt2 can signal through integrins, especially in low Tek conditions [7, 8]. Although we did not find any differences in expression of Angpt2 or Tek in at baseline in *Angpt1* ^*Δ/Δ*^, we investigated FAK phosphorylation at Tyr397 as a downstream target of integrin signaling [8]. Lungs from WT and *Angpt1* ^*Δ/Δ*^ at baseline and 4 h after tail vein injection of B16F10 melanoma cells showed no differences in FAK phosphorylation at Tyr397 (Fig. 4g).

An increased attachment of tumor cells to the vessel wall could be another explanation for an increased seeding of B16F10 cells to the lung in *Angpt1* ^*Δ/Δ*^ mice. To investigate this we performed in vitro experiments of the attachment of B16F10 melanoma cells to endothelial cells (HUVEC) with and without Angpt1 present. In these experiments we found that B16F10 cells attached significantly less to HUVEC treated with Angpt1 (Fig. 4h).

### Angiopoietin-1 does not affect tumor cell migration

**Fig. 5:**
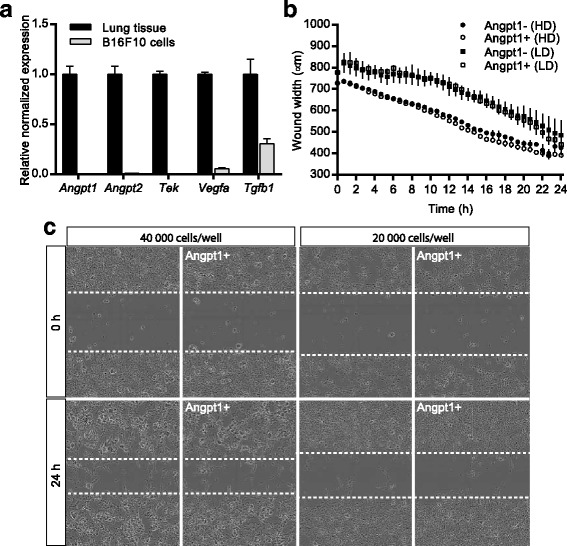
Effect of Angpt1 on B16F10 cells. **a** Expression analysis of *Angpt1*, *Angpt2* and *Tek* in B16F10 melanoma cells compared to lung tissue. **b**-**d** Quantification of migration assays (**b**) and representative images (**c**) of B16F10 cells in the presence or absence of Angpt1. In (**b**) every second value vas excluded for clarity. HD – high density, 40,000 cells/well, LD – low density, 20,000 cells/well

Although the aforementioned experiments indicate that the increased metastasis is probably due to differences in the blood vessel physiology in Angpt1 knockout mice, we also wanted to exclude the possibility that tumor cells are affected. Using quantitative RT-PCR, we found that wildtype B16F10 cells do not express *Angpt1*, *Angpt2* and *Tek* comparing to whole lung from adults (Fig. 5a). Nevertheless, we still went ahead to investigate if their migration could be affected by Angpt1. We performed in vitro migration studies using a scratch/wound assay with two different densities of B16F10 cells. We found no difference in cell behavior for cells treated with Angpt1 compared to non-treated cells (Fig. 5b, c).

To investigate if mammary tumor cells from the PyMT mice express components of the Angpt1 system and we extracted data from a published RNA-seq study of PyMT mammary tumors. The late carcinoma stage mammary gland samples had a > 90% purity for tumor cells [25] and the expression was compared to mammary gland from FVB mice. Several genes had a lower expression in the PyMT tumors compared to FVB controls (Table 1). Notably, the expression levels of Tek are 6-fold lower in the PyMT model, suggesting that loss of expression might be important for tumor progression. However, this observation does not explain the enhanced metastasis in the case of B16F10 cells.

**Table 1:** RNA-seq of mammary tumors

Gene	Sign. Change	FVB	PyMT
*Angpt1*		270	210
*Angpt2*	↓	146	30
*Angpt4*		0	0
*Angptl1/Angpt3*	↓	46	6.3
*Angptl2*	↓	2430	308
*Angptl3*		6.0	0
*Angptl4*	↓	2138	341
*Angptl6*		12	9.3
*Angptl7*		0	1.3
*Tek/Tie2*	↓	604	96
*Tie1*	↓	416	105
*Vegfa*	↓	1621	574
*Kdr/Vegfr2*	↓	1483	230
*Flt/Vegfr1*	↓	1243	202
*Cxcl12*	↓	2241	363
*Cxcr2*		1	2
*Cxcr3*		19	2
*Cxcr4*		145	290

An extract of data from a published data set with RNA sequencing performed on mammary glands from FVB (control) and FVB mice with PyMT mammary tumors with late stage carcinoma [25]. Data are expressed as the average FPKM (*n* = 4/group) and significant differences are indicated with arrows up (↑) or down (↓) compared to WT

### *Angpt1*^*Δ/Δ*^ lungs exhibit similar profile of the other angiopoietins as WT lungs

We also wanted to exclude that Angpt1 knockout changes other angiopoietins and angiopoietin-like genes, thus we extracted RNA-seq data from an unpublished data set of adult lung tissue from WT and *Angpt1* ^*Δ/Δ*^. As expected, Angpt1 was lost in *Angpt1* ^*Δ/Δ*^ lung, but other angiopoietins and receptors were unchanged (Table 2.) In contrast, we found that the expression of some chemokines was altered. While increase production of Cxcl12 is a well-known mechanism that promotes distant metastasis, in the *Angpt1* ^*Δ/Δ*^ we observed a slight decrease.

**Table 2:** RNA-seq of lung tissue in WT and *Angpt1* ^*Δ/Δ*^ mice

Gene	Sign. Change	WT	Angpt1KO
*Angpt1*	↓	22.0 ± 6.2	0.5 ± 0.1
*Angpt2*		16.6 ± 0.5	14.4 ± 2.1
*Angpt4*		0.4 ± 0.09	0.5 ± 0.11
*Angpl1/Angpt3*		1.6 ± 0.15	1.7 ± 0.72
*Angptl2*		35.8 ± 2.7	34.0 ± 0.9
*Angptl3*		0.9 ± 0.4	0.8 ± 0.1
*Angptl4*		8.7 ± 0.4	13.9 ± 2.0
*Angptl6*		1.2 ± 0.4	1.8 ± 0.3
*Angptl7*		1.2 ± 0.3	1.5 ± 0.5
*Tek/Tie2*		144 ± 12	130 ± 4
*Tie1*		57 ± 2	57 ± 4
*Vegfa*		437 ± 47	509 ± 39
*Kdr/Vegfr2*		138 ± 8	146 ± 2
*Flt/Vegfr2*		85 ± 5	82 ± 4
*Icam1*		215 ± 6	215 ± 8
*Icam2*		89 ± 10	93 ± 7
*Vcam1*		38 ± 1	36 ± 1
*Cxcl12*	↓	77 ± 5	61 ± 2
*Cxcr2*	↓	7.0 ± 0.6	4.7 ± 0.2
*Cxcr3*	↑	0.94 ± 0.18	1.77 ± 0.20
*Cxcr4*		24.5 ± 1.6	23.1 ± 0.5

An extract of data from RNA sequencing performed on lung tissue from adult WT mice (*n* = 3) and *Angpt1* ^*Δ/Δ*^ mice (*n* = 4). Data are expressed as average FPKM ± SEM. Arrows indicate significant differences up (↑) or down (↓) compared to WT

## Discussion

In the current study we investigate both primary tumor growth and metastasis in Angpt1 deficiency. We found that primary tumor growth is not affected in Angpt1 deficient mice utilizing both the spontaneous MMTV-PyMT mammary tumor model and subcutaneous flank injections of B16F10 melanoma cells.

The metastatic process comprises several events, including tumor invasion, intravasation of tumor cells, their circulation and arrest in capillary beds, extravasation into the distant organ and colonization [31]. In the current study, experiments with MMTV-PyMT mice allowed for auditing of tumor growth and metastasis in a spontaneous model. Angpt1 knockout MMTV-PyMT mice showed a significant increase in metastasis to the lung compared to control MMTV-PyMT mice, without affecting primary tumor growth (Fig. 1). The MMTV-PyMT model provides a reliable model of human disease and progression from noninvasive to invasive cancer [32]. Although a good model, there are some challenges when breeding it to a transgenic system for conditional knockout. Firstly, the onset of tumors is strain and gender dependent, thus the Angpt1 knockout mice was backcrossed to FVB for >6 generations and only females were used for the experiments. In this model, it is also difficult to investigate which events in the metastatic process that are affected in Angpt1 knockout leading to increased metastasis.

To study in more detail the later part of metastasis, i.e. attachment, rolling and extravasation, we performed tail vein injections of B16F10 melanoma cells and investigated their seeding to the lung 4 h, 24 h and 3 weeks after injection. We found that Angpt1 deficient mice had significantly more tumor cells in the lung at all time points (Figs. 2, 4).

In the experimental metastasis model, the wildtype B16F10 cells were injected in Angpt1 knockout mice. To rule out that the increased seeding of B16F10 cells in lungs of Angpt1 knockout mice depended on a change in the tumor cells themselves we performed some characterization studies of B16F10 cells. In vitro assays of B16F10 cells showed that were unresponsive to changes in Angpt1 concentration (Fig. 5b, c). In addition, B16F10 cells do not appear to express *Angpt1*, *Angpt2* and *Tek* (Fig. 5a).

Angpt1 has several well-known anti-inflammatory properties [33] while Angpt2 is pro-inflammatory and has been shown to activate Tek-positive tumor associated macrophages [12, 26]. We therefore investigated if changes in macrophages contributed to increased metastasis to the lung in Angpt1 deficient mice. We found no differences in macrophage polarization or differences in Tek-positive macrophage populations after tail vein injection of B16F10 melanoma cells (Fig. 4). The total number of macrophages was also similar; however, other inflammatory cells were not investigated.

To investigate the mechanism for the increased seeding to the lung in Angpt1 knockout mice we performed a number of experiments utilizing B16F10 cells. One explanation for an increase in lung seeding could be increased vascular leakage. We therefore investigated if blood vessels were leaky at baseline and after injection of B16F10 melanoma cells utilizing a small fluorescently labelled tracer, Cadaverin (1 kDa). Cadaverin is often used to investigate vascular leakage [34]. No increase in leakage was seen which is in accordance with previous studies at baseline in Angpt1 deficient mice [6, 22]. There are some limitations to this leakage method in our B16F10 injection model. The cadaverin is injected and allowed to circulate before tumor cells are injected. To remove intraluminal cadaverin the mouse is perfused with PBS followed by fixation. If tumor cells are clogging capillaries this would decrease the removal of intraluminal cadaverin and thus give a higher fluorescence signal for cadaverin in lung homogenates.

Cancer cells have been shown to slow down and arrest in capillaries of similar diameter as that of the tumor cells, suggesting that they first become physically restricted before forming stable attachments [35, 36]. The Angpt1/Tek system has been implicated in vessel diameter regulation. Goel et al. recently showed that inhibition of VE-PTP increased Tek activity and inhibited several stages of tumor progression and metastasis [15]. Apart from structural and functional normalization of tumor vessels, VE-PTP inhibition resulted in an increase in vessel diameter leading to improved tumor perfusion and reduced hypoxia [27]. Several studies in mice have shown an increase in vessel diameter when activating of Angpt1/Tek signaling through different methods [15, 29, 30]. Also, venous malformations are linked to a gain of function mutation in Tek [28]. Hence, it is possible that the loss of Angpt1 in our model would decrease capillary diameter and change the interaction between vascular endothelial cells and B16F10 cells, thus increasing attachment and extravasation. We therefore investigated cross-sectional area in electron micrographs of lung capillaries and found it to be similar in Angpt1 deficient mice and control mice (Fig. 4e). To further study if a smaller capillary diameter could explain the increased seeding of tumor cells to the lung we injected microspheres of similar size as the tumor cells (15 μm) intravenously. Microspheres showed a similar distribution in controls and Angpt1 deficient mice (Fig. 4f).

Angpt2 has been shown to induce endothelial destabilization through binding to β1-integrin in situations of low Tek expressions [7, 8]. Induced endothelial expression of Angpt2 leads to increased lung metastasis through reduced junctional Tek localization and increase β1-integrin signaling [13]. It should be noted that Angpt2 binds with a significantly higher affinity to Tek compared to integrins, perhaps explaining why low Tek may be required for Angpt2-integrin signaling to occur. In the current study, we could not find any changes in the expression of Angpt2 and Tek in *Angpt1* ^*Δ/Δ*^ mice, or any changes of FAK phosphorylation at Tyr397 at baseline or after tail vein injection of B16F10 cells (Table 2, Fig. 4g). This suggests that integrin signaling is not changed in *Angpt1* ^*Δ/Δ*^ mice, hence not the mechanism for the increase in lung seeding of B16F10 cells in.

Angpt2 promotes inflammation by inducing vascular leakage and by increasing the expression the adhesion molecules Icam1 and Vcam1 [12], while Angpt1 reduces leukocyte adhesion by reducing the same factors [33]. Experiments with HUVECs and attachment of B16F10 cells in vitro showed that the presence of Angpt1 decreased attachment of B16F10 cells to the endothelial cells (Fig. 4h). However, we could not find any differences in *Icam* and *Vcam* expression in lungs of Angpt1 knockout mice compared to WT mice (Table 2). Future studies are needed to characterize the expression of these molecules in the endothelial compartment of the lungs. If other adhesion factors could be important for attachment of B16F10 cells to the endothelium in regards to the Angpt/Tek system remains to be investigated.

## Conclusions

In the current study we have shown that global Angpt1 deficiency results in increased metastasis to the lung without affecting primary tumor growth. Interestingly, Angpt1 appears to mostly affect the late stages of the metastatic process, attachment and extravasation, suggesting that Angpt1-Tek signaling normally act as a gate-keeper in capillaries.

## Abbreviations

Angpt1: Angiopoietin-1
Angpt2: Angiopoietin-2
FAK: Focal adhesion kinase
MMTV-PyMT: Polyomavirus middle T (PyMT) oncogene under control of the mouse mammary tumor virus long terminal repeat (MMTV)
Tek: Receptor tyrosine kinase
VE-PTP: Receptor-type tyrosine-protein phosphatase beta
